# BNST_AV_^GABA^-PVN^CRF^ Circuit Regulates Visceral Hypersensitivity Induced by Maternal Separation in Vgat-Cre Mice

**DOI:** 10.3389/fphar.2021.615202

**Published:** 2021-03-19

**Authors:** Si-Ting Huang, Zhi-Jing Song, Yu Liu, Wen-Chen Luo, Qian Yin, Yong-Mei Zhang

**Affiliations:** ^1^ Jiangsu Province Key Laboratory of Anesthesiology, Xuzhou Medical University, Xuzhou, China; ^2^ Department of Anesthesiology, Xuzhou Municipal Hospital Affiliated with Xuzhou Medical University, Xuzhou, China

**Keywords:** visceral hypersensitivity, hypothalamic paraventricular nucleus, bed nucleus of stria terminalis, GABA neurons, corticotropin-releasing factor, maternal separation

## Abstract

Visceral hypersensitivity as a common clinical manifestation of irritable bowel syndrome (IBS) may contribute to the development of chronic visceral pain. Our prior studies authenticated that the activation of the corticotropin-releasing factor (CRF) neurons in paraventricular nucleus (PVN) contributed to visceral hypersensitivity in mice, but puzzles still remain with respect to the underlying hyperactivation of corticotropin-releasing factor neurons. Herein, we employed maternal separation (MS) to establish mouse model of visceral hypersensitivity. The neuronal circuits associated with nociceptive hypersensitivity involved paraventricular nucleus CRF neurons by means of techniques such as behavioral test, pharmacology, molecular biology, retrograde neuronal circuit tracers, electrophysiology, chemogenetics and optogenetics. MS could predispose the elevated firing frequency of CRF neurons in PVN in murine adulthood, which could be annulled via the injection of exogenous GABA (0.3mM, 0.2µl) into PVN. The PVN-projecting GABAergic neurons were mainly distributed in the anterior ventral (AV) region in the bed nucleus of stria terminalis (BNST), wherein the excitability of these GABAergic neurons was reduced. Casp3 virus was utilized to induce apoptosis of GABA neurons in BNST-AV region, resulting in the activation of CRF neurons in PVN and visceral hyperalgesia. In parallel, chemogenetic and optogenetic approaches to activate GABAergic BNST_AV_-PVN circuit in MS mice abated the spontaneous firing frequency of PVN CRF neurons and prevented the development of visceral hypersensitivity. A priori, PVN^CRF^-projecting GABAergic neurons in BNST-AV region participated in the occurrence of visceral hypersensitivity induced by MS. Our research may provide a new insight into the neural circuit mechanism of chronic visceral pain.

## Introduction

The irritable bowel syndrome (IBS) is a common functional disease characterized by chronic abdominal pain and abnormality of bowel movement (Thompson et al., 1999; Farmer and Aziz, 2013), with a morbidity of approximately between 7 and 21% worldwide (Lovell and Ford, 2012; Vich Vila et al., 2018). The pathogenesis of IBS involves visceral hypersensitivity, abnormal gastrointestinal motility, disordered brain-gut-microbiota axis activity, psychological comorbidities, etc. (Moloney et al., 2016; Melchior et al., 2018). Notably, visceral hypersensitivity is identified in 20–90% of patients with IBS (Azpiroz et al., 2007). However, the essential mechanisms underpinning visceral hypersensitivity in IBS still await further illumination.

Early life stress (ELS) is an adverse stressful event in the early stage of life, which predisposes multiple diseases in adulthood including IBS (De Kloet et al., 2005). Maternal separation (MS) is commonly adopted as an ELS model to investigate the underlying mechanisms of functional gastrointestinal and psychiatric disorders (O'Mahony et al., 2009). ELS is known to affect the development of synaptic plasticity and neural circuits (Sun et al., 2019). Our previous studies identified that corticotrophin-releasing factor (CRF) neurons in the paraventricular nucleus (PVN) and hypothalamic-pituitary-adrenal (HPA) axis were involved in the development of MS-induced visceral hypersensitivity (Zhang et al., 2016; Tang et al., 2017). The activation of PVN CRF neurons and HPA axis is regulated by a variety of mechanisms, such as glucocorticoid feedback (Kloet, 2013), excitatory activity of glutamate neurons (Gunn et al., 2013) and the inhibitory action of γ-aminobutyric acid (GABA) neurons (Cullinan et al., 2008). PVN is governed dominantly by GABAergic neurotransmission (Decavel and Van den Pol, 1990). Importantly, the GABAergic afferents onto PVN can reportedly play crucial roles in visceral hypersensitivity. Moreover, there is morphological evidence that the GABA synapses in medial parvocellular paraventricular hypothalamus (PVNmp) account for approximately half of all synapses (Decavel and Van den Pol, 1990), wherein PVNmp cells receive GABAergic inhibitory inputs mainly from the marginal structures, including the medial preoptic area, the bed nucleus of the stria terminalis (BNST), and the medial hypothalamic nucleus (Ulrich-Lai and Herman, 2009). Therefore, the revelation of the upstream inhibitory nucleus projecting to PVN CRF neurons is of significance.

BNST is recognized as “extended amygdala, ”which connects the forebrain to the hypothalamus and brainstem regions and is associated with autonomic and neuroendocrine functions due to its remarkable effects on mood and emotion (Carlos et al., 2013; Lawrence, 2018). During the early postnatal period, BNST also undergoes developmental maturation like PVN CRF neurons, ELS renders it vulnerable to visceral hypersensitivity, anxiety-like and social behaviors, and so on (Emmons et al., 2021). BNST per se is an area with high heterogeneity in structure and function (Prewitt and Herman, 1998), with a vast majority of PVN CRF neurons receiving signals from its distinctive subregions. BNST is divided into the anterior and posterior regions, with the anterior area further divided into anterior middle (AM), anterior lateral (AL) and anterior ventral (AV) regions (Goodson and Kabelik, 2009; Davis et al., 2010). The vast majority of neurons in BNST-AV was GABAergic (Sun and Cassell, 1993), and most of GABAergic BNST-PVN projections are derived from BNST-AV GABAergic neurons to PVNmp, especially CRF neurons (Dong and Swanson, 2004; Dong and Swanson, 2005). MS can reportedly inhibit the activation of BNST neurons accompanied by decreased c-Fos expression (Banihashemi et al., 2011), whereas temporary MS suffices to evoke Fos expression within BNST (Fenoglio et al., 2006). In this respect, further exploration of the potential roles of BNST targeting visceral hypersensitivity instigated by MS would be of significance.

We hypothesized that inhibition of PVN-projecting GABAergic neurons in BNST-AV region could lead to the activation of PVN CRF neurons and the consequent visceral hypersensitivity in MS mice. To specifically validate the implication of GABAergic neurons in BNST_AV_-PVN circuit, vesicular GABA transporter (Vgat)-Cre transgenic mice were recruited in this study.

## Materials and Methods

### Animals

Vgat-ires-Cre knock-in mice (JAX number: 028862) aged 8–10 weeks were provided by the Jackson Laboratory in the United States. Vgat-Cre mice have Cre recombinase expression directed to inhibitory GABAergic neuron cell bodies for studying regulation of function or mapping the GABAergic neurons. Neonatal mice which were bred from the adult mice (one male with two females) until weaning at postnatal 21st day were randomly allotted in a standard triangular Plexiglas cage. All mice were housed under a 12h/12h light/dark cycle with food and water available ad libitum. All experiments were fully compliant with the National Institutes of Health Guidelines for the Care and Use of Laboratory Animals (NIH Publication No. 8023, revised 1978), and were approved by the Ethics Committee on Experimental Animal of Xuzhou Medical University.

### Animal Experimental Groups

The neonatal mice were divided into non-maternal separation (NMS) group and maternal separation (MS) group according to Miranda et al. (Armando et al., 2013). Owing to the difficulty of gender identification, all the pups in MS group were separated from the dams for 6h every day as of the 2nd to 15th day after birth (9:00–12:00 a.m. and 15:00–18:00 p.m.). Albeit female animals could serve to mimic the common clinical symptoms in female IBS-patients, the variations of estrogen and progesterone in response to CRD may affect the pain perception and underlying pain circuitry during the estrus cycle. In addition, BNST is a sexually dimorphic structure rich in distinctive neuronal subpopulations (Zhang et al., 2018; Liu et al., 2019). Accordingly, we only recruited neonatal male mice, which were reared to the postnatal 8^th^ week for experimentation.

### Reagents

Sheep polyclonal anti-Corticotropin Releasing Factor Antibody (NB110-81721, Novus, USA); rabbit anti-c-Fos mAb (2250s, Cell Signaling Technology, MA, USA); γ-aminobutyric acid (A2129-25G, Sigma-Aldrich LC, USA); muscimol hydrobromide (G019, Sigma-Aldrich); mouse monoclonal Anti-GAD 67 (MAB5406, Millipore, USA); rabbit polyclonal anti-GAD 67 (10408-1-AP, Proteintech, USA); mouse polyclonal anti-GAD 65 (20746-1-AP, Proteintech, USA); mouse anti-β-actin mAb (sc-47778, Santa Cruz, CA, USA); rabbit anti-GAPDH pAb (AC001, Abclonal, MA, USA); Alexa Fluor 488 donkey anti-Rabbit IgG (H + L) and Alexa Fluor 594 donkey anti-Sheep IgG (H + L) (Life Technologies, CA, USA); DAPI staining solution (C1005, Beyotime, China); alkaline phosphatase horse anti-mouse IgG (ZB-2310, Zsbio, China); HRP-labeled goat anti-mouse IgG (H + L) (A0216, Beyotime, China); BCA protein assay kit (P0012 Beyotime, China); RIPA lysis buffer (P00138, Beyotime, China); phenyl-methanesulfonyl fluoride (PMSF) (ST506, Beyotime, China); sodium dodecyl sulfate (SDS)-polyacrylamide gel electrophoresis (PAGE) sample loading buffer (P0015, Beyotime, China); BCIP/NBT alkaline phosphatase color development kit (C3206, Beyotime, China); BeyoECL Moon kit (P0018, Beyotime, China).

### Adeno-Associated Virus (AAV) Tools

PVN-microinjection: rAAV-CRF-EYFP-WPRE-pA (2.04 ×10^⁠12^ vg/ml, Customized, BrainVTA, China) ; rAAV-Ef1α-DIO-mCherry-WPRE-pA (retro) (2.06 ×10⁠^12^ vg/ml, BrainVTA, China).

BNST-microinjection: rAAV-Ef1α-DIO-mCherry-WPRE-pA (2.06×10⁠^12^ vg/mL, BrainVTA, China); rAAV-flex-taCasp3-TEVp-WPRE-pA, AAV 2/9 (2.06 ×10⁠^12^ vg/mL, BrainVTA, China). rAAV-Ef1α-DIO-hM4D(Gi)-mCherry-WPRE-pA (2.06×10⁠^12^ vg/ml, BrainVTA, China); rAAV-Ef1α-DIO-hM3D(Gq)-mCherry-WPRE-pA (2.06×10⁠^12^ vg/ml, BrainVTA, China); rAAV-Ef1α-DIO-hChR2(H134R)-mCherry-WPRE-pA (2.06 ×10⁠^12^vg/ml, BrainVTA, China); rAAV-Ef1α-DIO-eNpHR3.0-mCherry-WPRE-pA (2.06×10⁠^12^ vg/ml, BrainVTA, China).

A duration of two or three weeks was adequate for the AAV expression. The microinjection volume was 200nl unilaterally at a rate of 60nl/min.

### Detection of visceral Pain Threshold and Abdominal Withdrawal Reflex (AWR) Scores

For detection of visceral pain threshold as per the description by Julie et al. (Christianson and Gebhart, 2007), mice in both MS and NMS groups were anesthetized under sevoflurane, followed by insertion of an uninflated balloon coated with the paraffin oil into the colorectal tract, with the end of the balloon maintained 0.5cm away from the anal margin. After 15min of acclimatization, graded distension was performed by rapid inflation of the balloon to a pressure as specified values (20, 40, 60, 80mmHg) for 20s, followed by a 4min break. According to the response of colorectal walls of the mice to the expansion pressure, it is divided into the following four grades, i.e., AWR score: 0 point, no significant behavioral change; 1 point, motionless or only simple head movement; 2 points, contraction of abdominal wall muscles and contact with the table; 3 points, contraction of abdominal wall muscles and without contact with the table; 4 points, arching of abdominal wall with or without the arching of the body and pelvis. The pain threshold was determined as per the AWR scoring criteria, i.e., the minimum pressure value to induce significant contraction of the abdominal wall or lifting off the desktop (AWR score ≥3 points) recorded. Mice measurements were in triplicate and averaged for further analysis.

### High Plus Maze

The elevated cross maze experiment was mainly adopted to observe the anxiety state of animals. In brief, the elevated cross maze is 50cm high from the ground, consisting of a vertically crossed open arm (60cm × 5cm, no border) and a closed arm (60cm × 5cm, high border 25cm) composed of two arms, with a central cross area of 5cm × 5cm in the middle. Each mouse was allowed a duration of 5min for measurement. Caution should be taken to avoid noise and light stimulation. The murine subjects were placed in the middle of the platform, simultaneously ensuring that the head of the mouse was positioned toward the closed arm. An entry was defined as placement of the four paws within boundaries of the arm. The test process was recorded by a camera connected to the computer throughout the experiment, with the times of entry into the open arm recorded.

### Open Field Test

The device for the open field experiment was a box (50cm × 50cm × 45cm) with a white bottom evenly divided into 9 squares. The middle square was named as the central area and the ambient 8 squares outer area. Briefly, at the commencement of each experiment, the mice were gently placed in the central area. With the camera connected to the computer, the system was subsequently clicked on to start recording of mouse behavior within 5min. The observation index was: the percentage of the duration of mice in the central area.

### Sucrose Preference Test

The sucrose preference assay can reflect the euphoria of mice. The decreased consumption of sucrose indicated dysphoria in mice, which was one of the key symptoms of depression. The mice were deprived of water for 24h and subsequently underwent sucrose preference assay. During the experiment, each mouse was housed separately and provided with two drinking bottles containing 1% sucrose solution and pure water. Both bottles of liquid were weighed and recorded in advance. During the test, the positions of two bottles were exchanged. After 24h, the two bottles were retrieved for weighing to calculate the ratio of sucrose consumption of each mouse within 24h according to the following formula: sucrose consumption percentage = sucrose consumption/(sucrose consumption + pure water consumption) × 100%.

### Forced Swimming Test

Mice were gently placed in a glass tank (30cm in height, 15cm in diameter, 15cm in water depth), with the water temperature maintained at 25–30°C. Afterwards, the latency in which the mice struggled desperately to escape was recorded, and the duration in which the mice presented a typical "floating state" was recorded within 5 min, wherein "floating state" was defined as the motionless state of the mouse other than some necessary movement to keep the head above water. At the end of each test, fresh water was replaced to prevent the next mouse from being affected by the odor of the previous one and from infection from suffocation in water.

### Catheterization and Stereotactic Administration via Cannula in PVN

Adult male Vgat-Cre mice, 23–28g in weight, were anesthetized with 1% pentobarbital sodium (60mg/kg, i.p.). According to the Atlas of Mouse Brain by Keith BJ Franklin and George Paxinos, the third edition, coordinates for localization were: PVN (A/P: −0.05–0.15 mm; L/R: ± 0.25 mm; D/V: −5.0–5.1mm), BNST (A/P: 0.9–1.0 mm; L/R: ± 0.8 mm; D/V: −4.7–4.75mm). Each mouse was injected at a constant rate of 60nl/min, with the needle remaining in site for 10min thereafter to prevent the drug reflux. The experiment would be conducted in the case of the virus expression for at least 14 or 21 days.

PVN catheterization was performed as follows: with the mouse fixed on the brain stereotaxic instrument as described above, the 5.0mm ferruled cannula should be vertically fixed on the holder of the locator with the length of the inserted core needle slightly longer than that of the cannula. The dental cement was mixed around the sleeve and the metal cap was screwed onto the cannula. After 1 week, mice were anesthetized with inhaled sevoflurane of low concentration, the metal cap of the cannula was unscrewed, and the catheter inserted into the cannula, followed by gentle administration of the drug via a micro-syringe pump. The catheterization of optical fiber was identical to the procedures described above.

### Immunofluorescence Analyses

After deep anesthesia, the mice were transcardially perfused with 20ml of 0.9% saline, followed by infusion of 20ml 4% paraformaldehyde. The mouse brain was carefully isolated and further stored in 4% in polyoxymethylene at 4°C overnight for fixation before transference to 30% sucrose. The brain tissue was sectioned into 30μm-thick slices, and collected in 0.01M PBS. Slices were rinsed with PBS in triplicate (5min each), and blocked with 10% donkey serum for 2h prior to incubation with the c-Fos antibody (1: 1000) and CRF antibody (1: 200) diluted in PBS containing 0.3% Triton X-100 at 4°C for 24h. After PBS lavage, the corresponding sections were incubated with anti-mouse Alexa Fluro 488 or anti-rabbit Alexa Fluor 594 in dark at room temperature (r/t) for 2h. The tissue sections were counterstained with DAPI (4,6-diamino-2-phenylindole) before mounting with 90% glycerol and visualized with a confocal laser microscope (FV1000, Olympus, Tokyo, Japan).

### Western Blot Analysis

Western blot analysis was adopted to characterize the protein expression in PVN and BNST-AV. Mice in each group were quickly decapitated to isolate brains and obtain target tissues. The brain tissues were placed into a 2ml pre-chilled Eppendorf tube containing RIPA lysis buffer with PMSF. The specimens were centrifuged at 12000rpm for 15min at 4°C, followed by collection of supernatants. 15μg of protein lyses was separated with 10% separation polyacrylamide gel before transference onto the PVDF membrane. With the addition of 5% skim milk and storage on a shaker for 2h for blockage, the membranes were incubated with primary antibody at 4°C in a shaker overnight. The primary antibodies included: mouse anti-CRF primary antibody (1: 1000), mouse anti-GAD65 and GAD67 primary antibodies (1: 1000), mouse anti-GAPDH primary antibody (1: 1000) or mouse anti-β-actin (1: 1000). After triplicated lavage with washing buffer for 5min on the next day, the membranes were incubated with the corresponding secondary antibody at r/t for 40min on a shaker. After rinse in triplicate with washing buffer 10min, the protein band were visualized by ProPlus image analysis system (NIH, Bethesda, MD, United States) and analyzed by Image J software.

### *In vitro* Electrophysiology

Cell-attached recording is extensively adopted to study the spontaneous firing of mammalian neurons. PVN or BNST-AV neurons were recorded in brain slices via cell-attached electrophysiology. After anesthetization, each mouse was transcardially perfused, followed by isolation of mouse brain, which were to be sectioned into 280μm-thick slices. The sections were incubated in high-sugar cerebrospinal fluid at 34°C for 1h, and at r/t for another 30min. Then the brain slices were transferred to the perfusion tank on the electrophysiological table with a special pipette for recording. The brain slices were perfused with artificial cerebrospinal fluid (ACSF), which was fully oxygenated with a mixture of 95% O_2_ and 5% CO_2_. The composition of high-sugar cerebrospinal fluid was (mM): 3.5 KCl, 0.5 CaCl_2_, 4.5 MgSO_4_, 80 NaCl, 90 sucrose, 10 glucose, 1.25 NaH_2_PO_4_, 25 NaHCO_3_ (295–305 mOsm, pH = 7.35). The composition of ACSF was (mM): 2.5 KCl, 126 NaCl, 1.2 MgSO_4_, 1.2 NaH_2_PO_4_, 26 NaHCO_3_, 2.4 CaCl_2_ and 10 glucose (295–305 mOsm, pH 7.35). Microelectrode was immerged in the electrode solution (Pippette solution) and the electrode resistance was set to 6–10MΩ. The composition of the electrode solution was (mM): 10 HEPES, 5 EGTA, 135K gluconate, 2 MgCl_2_, 3 ATP-Mg, 0.5 CaCl_2_, 0.2 GTP-Na (280–290 mOsm, pH = 7.25). The sealing resistance was greater than 70MΩ for recording the spontaneous discharge. The parameter was set at the current clamp with I = 0, the high wave filtering was 300kHz, and the low wave filtering was 0.5kHz, the signal acquisition frequency was 10kHz, and the recording commenced after the onset of a signal with a regular discharge frequency.

In adult mice with MS-induced chronic visceral pain, rAAV-Ef1α-DIO-hChR2(H134R)-mCherry-WPRE-pA was bilaterally injected into the BNST-AV area, and rAAV- CRF-EYFP-WPRE-pA virus was bilaterally injected into the PVN. After 21 days, the blue light (473nm) was employed to activate the BNST-AV GABAergic neuron terminals extending to PVN. The depolarization current of the GABA neurons in the BNST-AV area was recorded. The discharge frequencies during light-on period and before and after blue light excitation were counted separately. In pharmacological experiments, the firing frequencies before and after exogenous GABA perfusion were separately calculated.

### Chemogenetics

rAAV-Ef1α-DIO-hM4D(Gi)-mCherry-WPRE-pA and rAAV-Ef1α-DIO-hM3D(Gq)-mCherry-WPRE-pA were bilaterally injected into the BNST-AV region. With respect to clozapine-N-oxide (CNO) administration in PVN, a trocar was implanted 0.2mm above the third ventricle adjacent to PVN (A/P: -0.05–0.15mm, L/R: ±0.25mm, D/V: -4.80mm from the bregma). Mice received PVN infusion of vehicle or CNO to stimulate the Gi/Gq-coupled designer receptor exclusively activated by designer drugs (DREADD). Behavioral experiments were performed at least 3 weeks thereafter to allow for sufficient viral expression.

### Optogenetic Experiment

rAAV-Ef1α-DIO-hChR2(H134R)-mCherry-WPRE-pA and rAAV-Ef1α-DIO-eNpHR3.0-mCherry-WPRE-pA were bilaterally injected into the BNST-AV region. For optogenetic manipulation, the optical fiber was implanted above the third ventricle adjacent to PVN (A/P: −0.05–0.15 mm, L/R: ± 0.25mm, D/V: −4.80mm from the bregma). The optical power was 1.8mW for the blue laser (473nm) and 3.5mW for the yellow laser (589nm), as measured at the tip of the optic fiber. The photogenetic electrophysiology of isolated brain slices revealed the frequency of 10Hz and the wave width of 10ms, and blue laser with a wave length of 473nm could cause ChR2-mCherry + neurons to produce depolarizing current. Meanwhile, we also utilized a blue light with a frequency of 10Hz and a wave width of 10ms to activate the BNST_AV_-PVN GABAergic neuron terminals, aiming to investigate the specific circuit effects on the visceral pain threshold. Mice underwent constant photostimulation for 10min, with the visceral pain threshold detected 5min after illumination of blue light pulses. The average value was calculated in triplicate for further analysis.

### Statistical Analysis

Data are expressed as mean ± S.E.M. Independent samples *t*-test was used between two groups; and one-way ANOVA was used among multiple groups. The time courses of pain thresholds were analyzed with a two-way repeated measure ANOVA. For statistical differences, post hoc Bonferroni or SNK was used for pairwise comparison. The test level was set as α = 0.05, and *p* value less than 0.05 was considered statistically significant.

## Results

### Maternal Separation in Neonatal Mice Induced Visceral Hypersensitivity, Anxiety-and Depression-like Behavior in Adulthood

**FIGURE 1:**
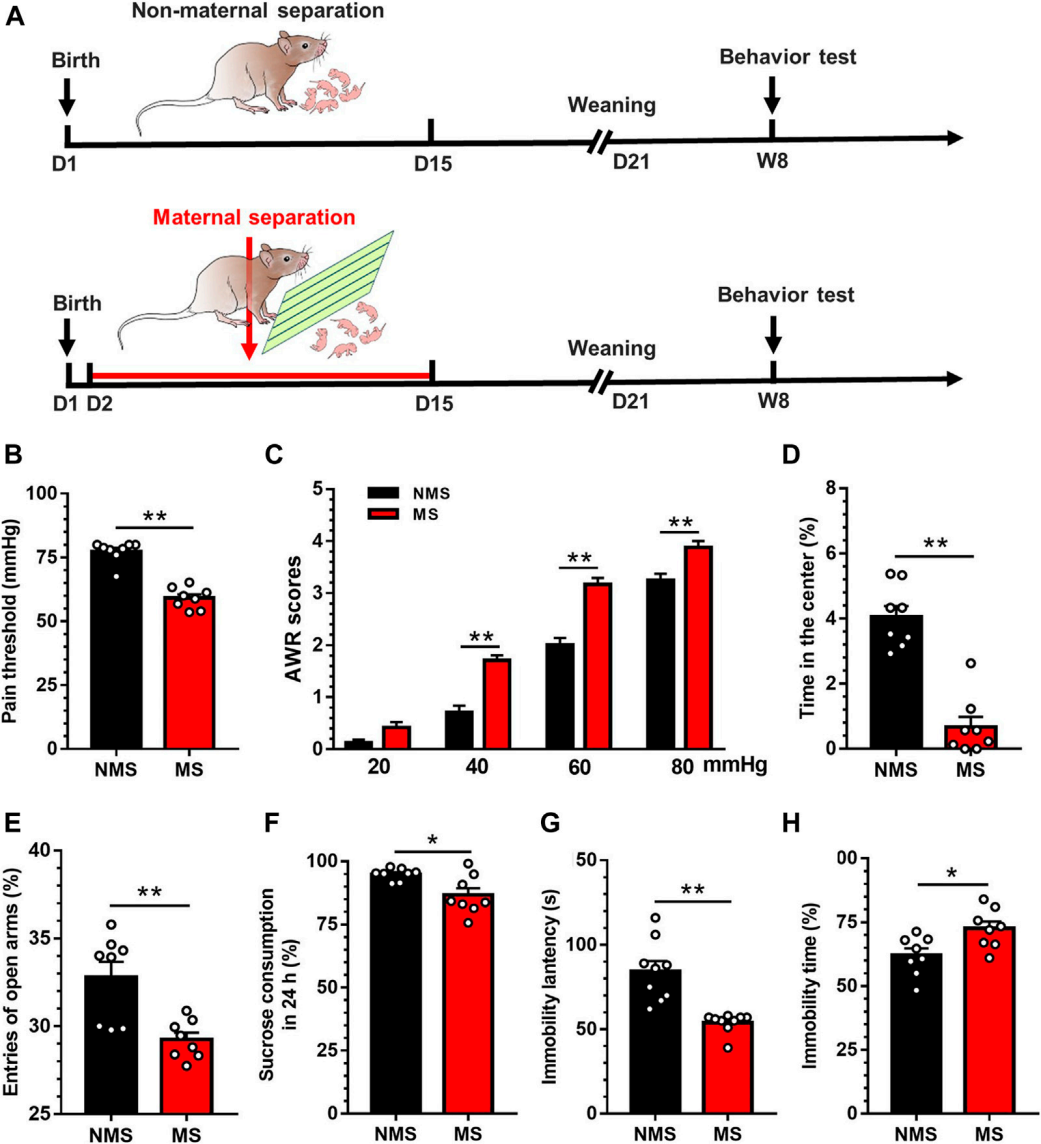
Chronic visceral pain, anxiety- and depression-like behaviors were induced by MS in mice **(A)** The establishment of maternal separation model **(B)** Visceral pain threshold in MS group was decreased compared with NMS group (*n* = 8) **(C)** AWR scores in MS group were increased compared with NMS group (*n* = 8) **(D)** Duration in the center was significantly decreased in MS group compared with NMS group (*n* = 8) **(E)** The times of entry into open arms was significantly decreased in MS group compared with NMS group (*n* = 8) **(F)** The sucrose consumption in MS group was significantly decreased compared with NMS group in 24h (*n* = 8) **(G)** The latency of immobility in MS group was significantly decreased in comparison with NMS group in forced swimming test (*n* = 8) **(H)** The immobility duration in MS group was significantly increased compared with NMS group in forced swimming test (*n* = 8). Data are presented as the mean ± S.E.M. *p < 0.05, **p < 0.01 compared with NMS group.

The timeline of model establishment and behavioral detection is depicted in Figure 1A. Neonatal mice were separated from the maternal mouse for 6h per day as of the 2nd–15th day postnatally. To minimize the confounders in measurements in mice, each group (*n* = 8 mice) only underwent a single item of the behavior experiments performed by individual technicians. In this respect, the time point of each behavior experiments could not have resulted in the bias which might have occurred in mice undergoing all the behavior experiments. In parallel, mice with NMS underwent all the procedures other than MS. Consequently, visceral pain threshold in the MS group was significantly lower than that in the NMS group[t (14) = 8.75, *p* < 0.01; Figure 1B]. Likewise, the elevated AWR scores further authenticated the development of visceral hypersensitivity in MS mice rather than NMS group (Figure 1C). A two-way repeated measures ANOVA revealed that a significant difference in group [F (1, 14) = 58.79, *p* < 0.01], treatment [F (3, 42) = 375.9, *p* < 0.01] and group × treatment interaction [F (3, 42) = 6.80, *p* < 0.01]. Post hoc Bonferroni multiple comparisons indicated a significant increase in the AWR scores at 40, 60 and 80mmHg in MS mice as compared with NMS group (*p* < 0.01). As for the open field test, the duration in the central area was significantly lower in the MS group than in the NMS group [t (14) = 7.38, *p* < 0.01; Figure 1D]. The times of entry into open arms in the MS group was significantly lower than that in the NMS group in the elevated maze test [t (14) = 3.71, *p* < 0.01; Figure 1E], suggesting that MS could induce anxiety-like behavior in adult mice. The sucrose preference assays showed that sucrose consumption was significantly reduced at 24h in the MS group compared with the NMS group [t (14) = 2.89, *p* < 0.05; Figure 1F)] Forced swimming test results presented shortened immobility latency and prolonged immobility duration in the MS group vs. the NMS group [t (16) = 4.81, *p* < 0.01; Figure 1G t (14) = 2.74, *p* < 0.05; Figure 1H], indicating that MS induced depression in adult mice. These results demonstrated that neonatal MS mice were susceptible to the visceral hypersensitivity and anxiety- and depression-like behaviors in adulthood.

### Involvement of Activation of CRF Neurons in PVN in the MS-Induced Visceral Hypersensitivity

**FIGURE 2:**
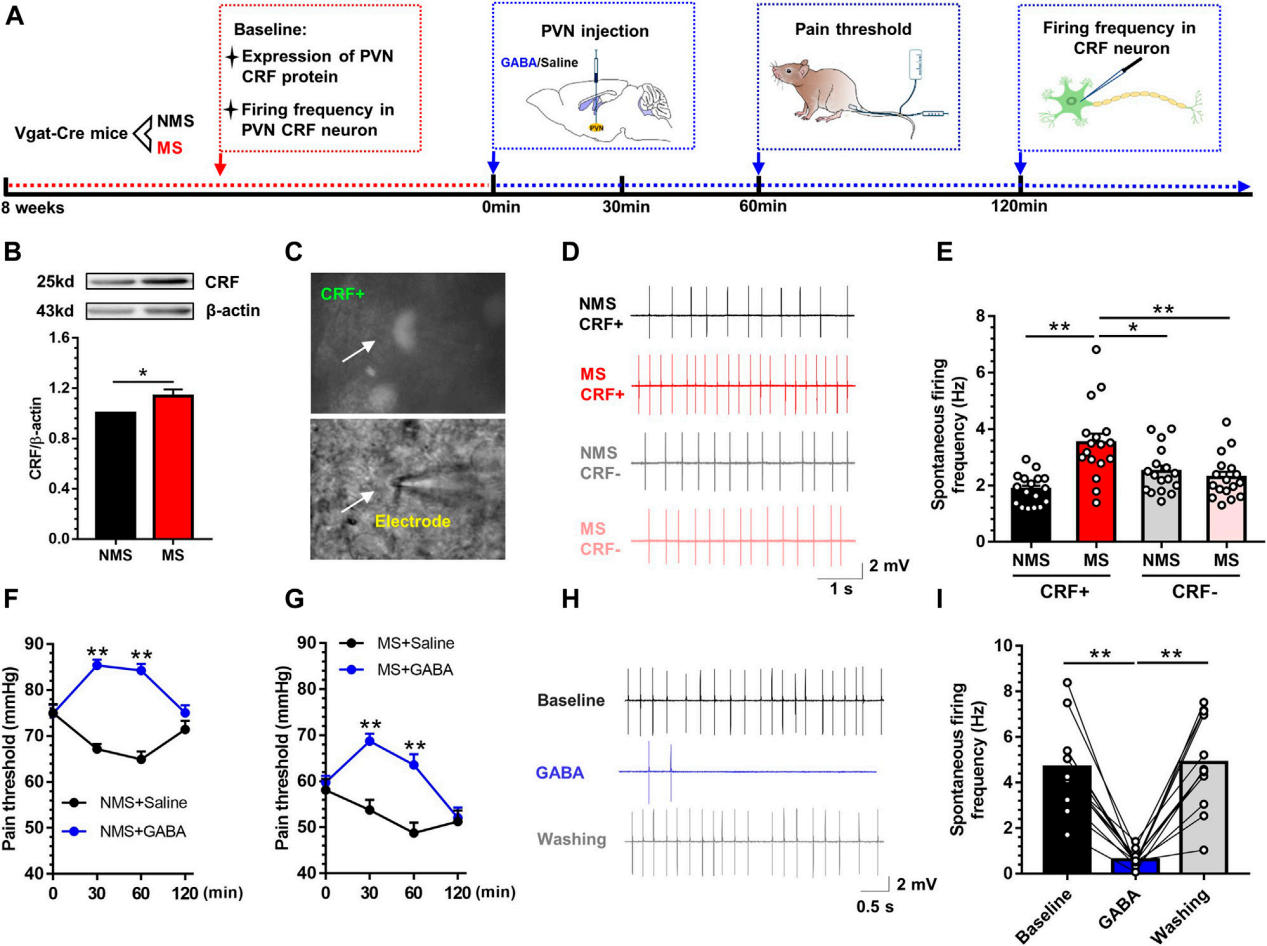
MS induced the activation of CRF neurons in PVN, while microinjection of exogenous GABA into PVN could alleviate the visceral pain **(A)** The experiment flow and timeline for the activity of PVN CRF neurons **(B)** Western blot showed that the expression of CRF protein was significantly increased in MS group vs. NMS group (*n* = 6) **(C)** The diagram of specific CRF-neurons **(D)** Schema of the typical firing frequency **(E)** The spontaneous firing frequency of CRF neurons in MS group was significantly increased vs. NMS group (*n* = 17, six mice per group) **(F)** Injection of exogenous GABA in PVN increased the visceral pain threshold in NMS mice vs. saline group (*n* = 8) **(G)** Injection of exogenous GABA in PVN increased the visceral pain threshold in MS mice vs. saline group (*n* = 8) **(H)** Schema of the typical firing frequency **(I)** Perfusion of exogenous GABA throughout brain slices could decrease the CRF neurons firing frequency in PVN of mice vs. baseline and post-perfusion profiles (*n* = 12, six mice per group). Data are presented as the mean ± S.E.M. *p < 0.05, **p < 0.01 vs. indicated group.

To explore the mechanism that may underlie the development of visceral hypersensitivity, we first evaluated whether MS could induce the activation of PVN CRF neurons. The experimental flow is shown in Figure 2A. Western blot revealed that the expression of CRF protein in the PVN was significantly upregulated in the MS group as compared to the NMS group [t (10) = 2.52, *p* < 0.05; Figure 2B]. Afterwards, electrophysiological patch clamp technique was utilized to record the firing frequency of CRF neurons in PVN. For the specific recognition of CRF neurons, CRF-specific promoter AAV virus (rAAV-CRF-EYFP-WPRE-pA) was injected into PVN 21 days preceding the visceral pain threshold measurement and electrophysiological recording. Figure 2C illustrated the schema of CRF labeling neurons. The schematic diagram of firing frequency is shown in Figure 2D. One-way ANOVA showed a significant difference in discharge frequency of CRF neurons in PVN [F (3,64) = 10.00, *p* < 0.01; Figure 2E)]. Post hoc Bonferroni multiple comparisons showed that the firing frequency of CRF neurons was significantly increased compared with other three groups. In our previous study (Song et al., 2020), GABA has been reportedly implicated in the pathogenesis of visceral hypersensitivity, and we referred to the PVN administration of exogenous GABA (0.3mM, 0.2µL). The visceral hypersensitivity was altered after GABA treatment in NMS mice (Figure 2F). A two-way repeated measures ANOVA showed that a significant difference in group [F (1, 14) = 11.82, *p* < 0.01], time [F (3, 42) = 13.59, *p* < 0.01] and group × time interaction [F (3, 42) = 12.41, *p* < 0.01]. Post hoc Bonferroni multiple comparisons showed that the visceral pain threshold was significantly increased 30 and 60min after injection of exogenous GABA compared with saline injection (*p* < 0.01). Similarly, the visceral hypersensitivity was relieved after GABA treatment in MS mice (Figure 2G). A two-way repeated measures ANOVA showed that a significant difference in group [F (1, 14) = 27.91, *p* < 0.01], time [F (3, 42) = 2.86, *p* = 0.048] and group × time interaction [F (3, 42) = 45.25, *p* < 0.01]. Post hoc Bonferroni multiple comparisons showed that the visceral pain threshold was significantly increased 30 and 60min after injection of exogenous GABA compared with saline injection (*p* < 0.01). Further, electrophysiological recording was adopted to assess the spontaneous firing frequency of CRF neurons in PVN in mice with chronic visceral pain induced by neonatal MS, wherein GABA (3μM) was dissolved with artificial cerebrospinal fluid (ACSF). The schematic diagram of firing frequency is shown in Figure 2H. Exogenous GABA inhibited the discharge frequency of CRF neurons in PVN in mice with neonatal MS. One-way ANOVA showed a significant difference [F (2,33) = 26.71, *p* < 0.01; Figure 2I]. Post hoc Bonferroni multiple comparisons showed that the firing frequency of CRF neurons was significantly decreased with GABA perfusion compared with baseline and post-administration lavage. These results demonstrated that the PVN CRF neurons were activated in mice experiencing MS.

### Distribution of GABAergic Neurons Projecting to PVN in the Anterior Ventral Area of BNST

**FIGURE 3:**
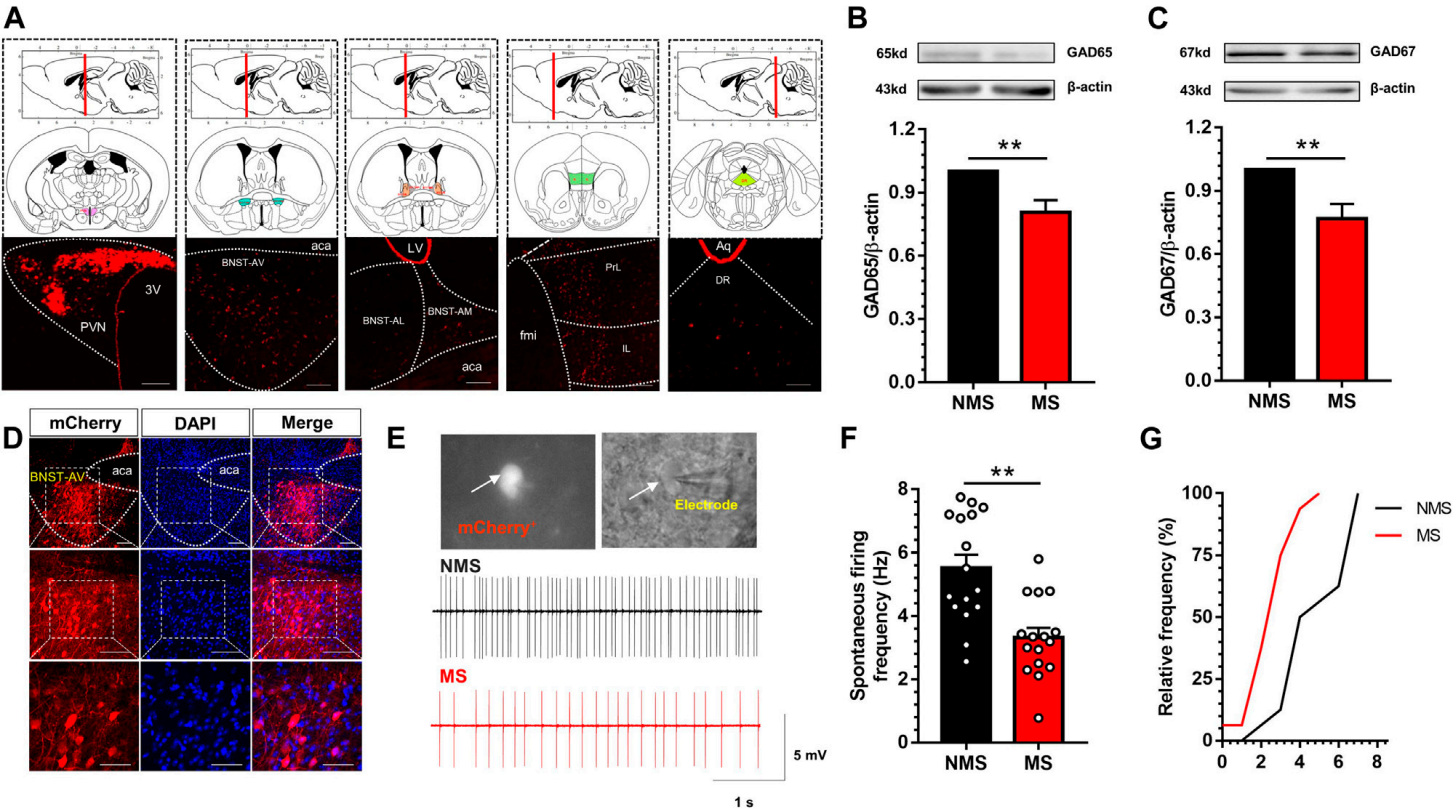
Excitation of GABA neurons in BNST-AV region was decreased in mice with MS-induced chronic visceral pain **(A)** Luma was absorbed by PVN neurons and directly transmitted to mPFC, BNST-AV, BNST-AL, BNST-AM and DR (3V, third ventricle; fmi, forceps minor of corpus callosum; PrL: prelimbic cortex; IL: infralimbic cortex; aca, anterior limb of ac; LV, lateral ventricle; Aq, mesencephalic aqueduct; DR: dorsal raphe nucleus). scale bar = 100μm **(B–C)** Western blot analysis of GAD65 and GAD67 protein in the BNST-AV demonstrated that MS mice presented significant decrease in the expression of GAD65 protein (B, *n* = 6; **p < 0.01) and GAD67 (D, *n* = 6; **p < 0.01) compared with NMS group **(D)** The localization of GABAergic BNST_AV_-PVN neurons via PVN-injected retrograde rAAV-Ef1α-DIO-mCherry-WPRE-pA in Vgat-Cre mice. Scale bar = 100μm except for the bottom marked by 50μm **(E)** The schema of spontaneous firing frequency in GABAergic neurons in BNST-AV regions **(F)** The spontaneous firing frequency of GABA neurons (mCherry+) in MS group was significantly decreased vs. NMS group (*n* = 16, six mice per group; **p < 0.01) **(G)** The cumulative frequency distribution curves in MS groups were evidently shifted leftward.

Accumulative studies have confirmed that PVN receives inhibitory neuronal projections mostly from BNST. In order to further identify and map the specific areas of neuronal distribution, we first traced the upstream nuclei projecting to PVN via PVN-injection of Lumafluor/Red retrobeads. We observed the red beads were absorbed by PVN neurons and directly transmitted to mPFC, BNST-AV, BNST-AL, BNST-AM and DR (Figure 3A). The red beads of BNST could be identified in BNST-AL, BNST-AM and BNST-AV, and were mainly concentrated in BNST-AV. After calculation, the proportion of red beads retrograde to BNST-AV region accounts for 72.7% of the total projection area in BNST. Thereafter, GABA synthetases GAD65 and GAD67 were employed to detect the expression of GABA in the BNST-AV region. GAD65 and GAD67 are glutamic acid decarboxylase necessary for the synthesis of GABA neurons, with GAD65 mainly distributed in the synaptic cleft, whereas GAD67 mainly identified in neurons. As a result, the expression of GAD65 protein in the BNST-AV area was significantly decreased in the MS group compared with the NMS group [t (10) = 3.34, *p* < 0.01; Figure 3B], as well as the expression of GAD67 [t (10) = 3.41, *p* < 0.01; Figure 3C]. Afterward, Cre-dependent retrograde virus rAAV-Ef1α -DIO-mCherry-WPRE-pA was injected into PVN, and the mCherry fluorescence was detected in BNST-AV 21 days thereafter (Figure 3D). Figure 3E described the schema of GABA neurons and discharge diagram of GABA neurons. Consequently, the spontaneous discharge frequency of GABA neurons was lower in the BNST-AV region in the MS group than in the NMS group [t (30) = 4.17, *p* < 0.01; Figure 3F], with the evidently leftward distribution curve of cumulative frequencies (Figure 3G). These data authenticated the exact existence of direct GABAergic BNST_AV_-PVN projection, and the attenuated excitability of GABA neurons in BNST-AV area projecting to PVN in the case of MS-induced visceral hypersensitivity.

### Ablation of BNST-AV GABAergic Neurons Induced the Activation of CRF Neurons in PVN and Visceral Pain

**FIGURE 4:**
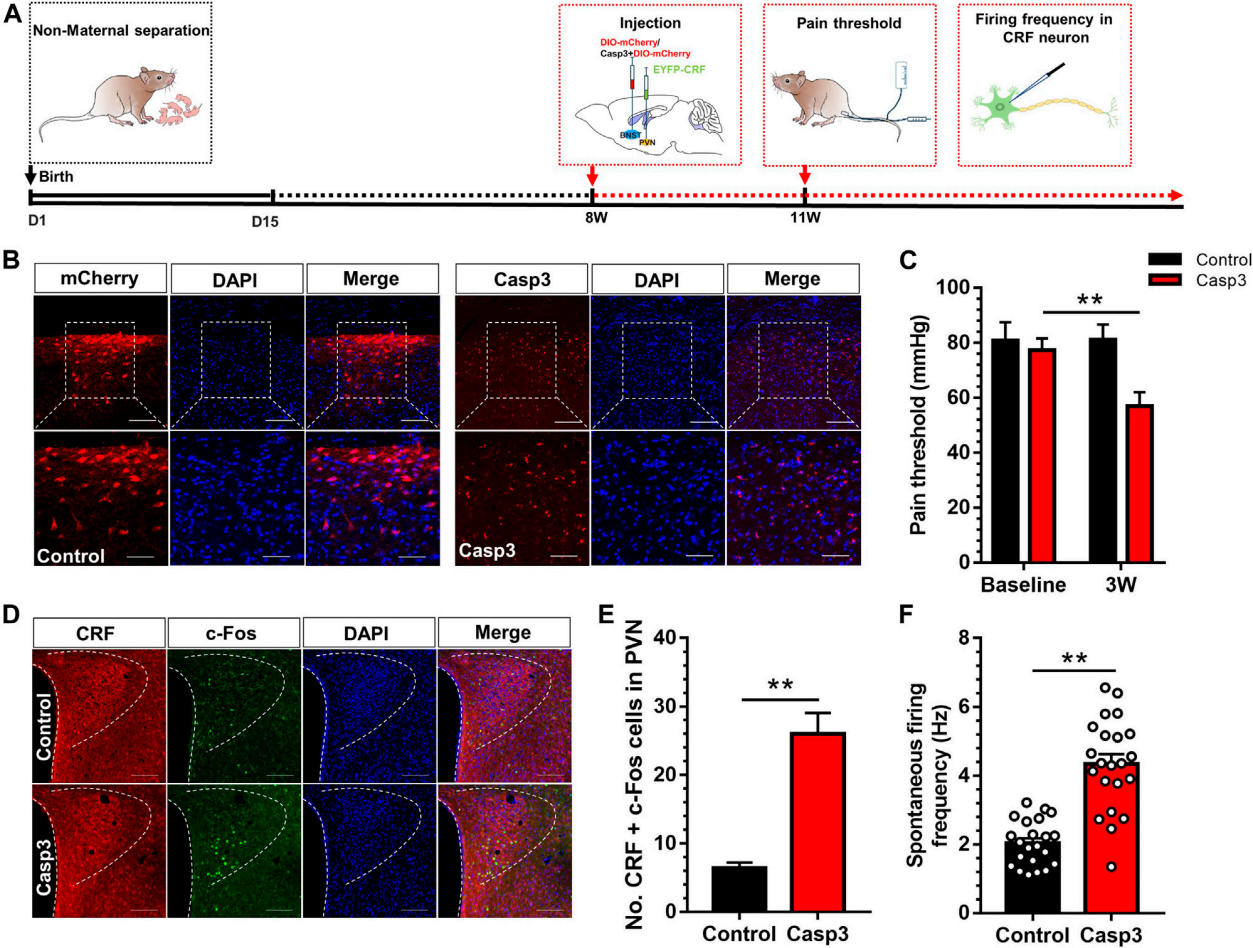
Destruction of GABA neurons in BNST-AV region resulted in the activation of CRF neurons in PVN and the reduction of visceral pain threshold **(A)** The timeline for virus injection and behavioral detection **(B)** Coronal section was monitored for Vgat-mCherry fluorescence and stained with DAPI 3 weeks after BNST-AV injection of Casp3, and the GABA neurons in the BNST-AV region of Casp3 mice exhibited alterations of cell morphology vs. mCherry group. The upper Scale bar = 100μm. The bottom Scale bar = 50μm **(C)** The visceral pain threshold was significantly decreased 3 weeks after Casp3 treatment vs. its baseline (*n* = 8, **p < 0.01) **(D)** Fluorescence image of anti-CRF and anti-c-Fos protein **(E)** The count of CRF neurons co-labeled with c-Fos was significantly increased in the PVN of Casp3 group vs. control group (*n* = 3, **p < 0.01) **(F)** The spontaneous firing frequency of CRF neurons (EYFP+) in Casp3 group was significantly increased vs. control group (*n* = 22, six mice per group; **p < 0.01).

Subsequently, a specific pro-apoptotic virus was injected in BNST-AV region to observe whether GABA neurons in BNST-AV region were involved in the regulation of visceral pain in normal Vgat-Cre mice. The virus can specifically damage the GABAergic BNST-AV neurons in Vgat-Cre transgenic mice by means of BNST-AV injection of rAAV-flex-taCasp3-TEVp-WPRE-pA, and the morphology of GABAergic neurons in BNST-AV were observed via PVN injection of retrograde rAAV-Ef1α -DIO-mCherry-WPRE-pA (hereinafter referred to as Casp3 group), whereas mice in the control group were treated with retrograde rAAV-Ef1 alpha -DIO-mCherry-WPRE-pA (control group). The time flow of apoptotic experiment is shown in Figure 4A. The apoptotic effect of GABA neurons in the BNST-AV area could be verified by loss of original cellular morphology (Figure 4B). Moreover, the visceral hyperalgesia developed in the Casp3 group compared with the control group (Figure 4C). A two-way repeated measures ANOVA showed that a significant difference in group [(F (1, 14) = 43.41, *p* < 0.01], time [F (1, 14) = 32.77, *p* < 0.01] and group × time interaction [F (1, 14) = 34.14, *p* < 0.01]. Post hoc Bonferroni multiple comparisons showed that the visceral pain threshold in Casp3 group was significantly decreased three weeks after microinjection (*p* < 0.01). Destruction of these regions will cause irreversible “neuronal death”. Moreover, the visceral pain threshold in Casp3 group was still decreased 2 months after microinjection, and we speculated this effect will persist in the absence of a compensatory mechanism. Further, the activity of CRF neurons was detected to explore the regulatory role of GABAergic neurons in BNST_AV_-PVN region. The colocalization of c-Fos and CRF in PVN was delineated in Figure 4D, and the population of c-Fos-labeled CRF neurons significantly increased after Casp3 virus treatment [t (8) = 6.19, *p* < 0.01; Figure 4E]. Likewise, the firing frequency of CRF neurons in PVN was significantly increased in Casp3 group compared with the control group (Figure 4F). These findings indicated that destruction of GABAergic BNST-AV neurons facilitated the activation of CRF neurons in PVN and the development of visceral hypersensitivity.

### Inhibition of GABAergic BNST_AV_-PVN Neurons Facilitated the Visceral Pain

**FIGURE 5:**
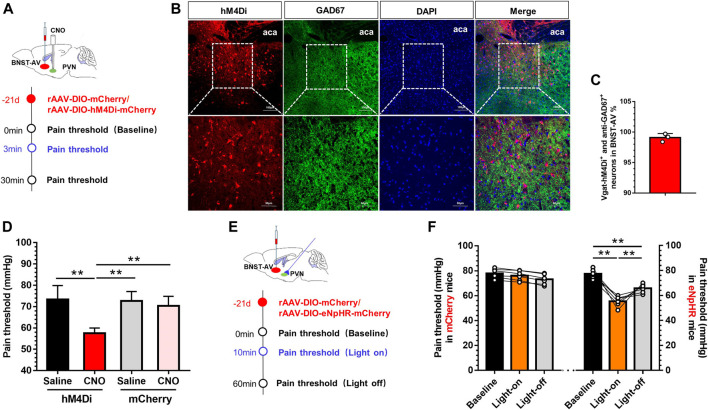
Inhibition of GABAergic BNST_AV_-PVN neurons induced the visceral pain in normal Vgat-Cre mice **(A)** Schematic diagram of AAV-DIO-hM4Di-mCherry injection into the BNST-AV and trocar implantation into the PVN in Vgat-Cre mice **(B)** The Vgat-hM4Di-mCherry neurons were stained with antibody to GAD67 to verify effects of virus-labeled GABAergic neurons in the BNST-AV **(C)** The co-standard rate of labeled GABAergic neurons and anti-GAD67 protein were 99.1% **(D)** The visceral pain threshold was significantly decreased post-CNO PVN injection (*n* = 6, **p < 0.01) **(E)** Schematic diagram of AAV-DIO-eNpHR-mCherry injection into the BNST-AV and fiber-optic incannulation into the PVN in Vgat-Cre mice **(F)** The visceral pain threshold was significantly decreased after photoactivation of the terminal of PVN-projecting eNpHR-mCherry-positive neurons from BNST-AV area (*n* = 6, **p < 0.01).

Next, we adopted chemogenetic manipulation to silence the PVN-projecting BNST-AV GABAergic neurons. Figure 5A illustrated the experimental schema of bilateral infusion of AAV-DIO-mCherry or AAV-DIO-hM4Di-mCherry into the BNST-AV and incannulation above PVN in normal Vgat-Cre mice. Confocal image of hM4Di and GAD67 in BNST-AV area was shown in Figure 5B. 99.1% hM4Di-tagged Vgat neurons were GAD67-inmmue-positive (Figure 5C). In Vgat-Cre mice, intra-PVN administration of CNO facilitated the visceral hypersensitivity (Figure 5D). One-way ANOVA showed a significant difference [F (3,20) = 14.89, *p* < 0.01; Figure 2E]. Post hoc Bonferroni multiple comparisons showed a significant decrease in the visceral pain threshold compared with other three groups (*p* < 0.01). Furthermore, optogenetic technique was employed to validate the effects on the PVN-projecting BNST-AV GABAergic neurons. The virus strategy was shown in Figure 5E. Consistent with above results, optogenetic inhibition (593nm) of terminals of neurons projecting to GABAergic BNST_AV_-PVN decreased the visceral pain threshold and facilitated pain (Figure 5F). One-way ANOVA showed a significant difference [F (2,15) = 50.54, *p* < 0.01; Figure 5F]. Post hoc Bonferroni multiple comparisons showed a significant decrease in the visceral pain threshold of eNpHR group compared with vehicle group (*p* < 0.01). These findings implied the important role of PVN-projecting GABAergic neurons in BNST-AV region in the modulation of visceral hypersensitivity.

### Activation of GABAergic BNST_AV_-PVN Neurons Inhibited the Activation of CRF Neurons in PVN and Alleviated Visceral Pain

**FIGURE 6:**
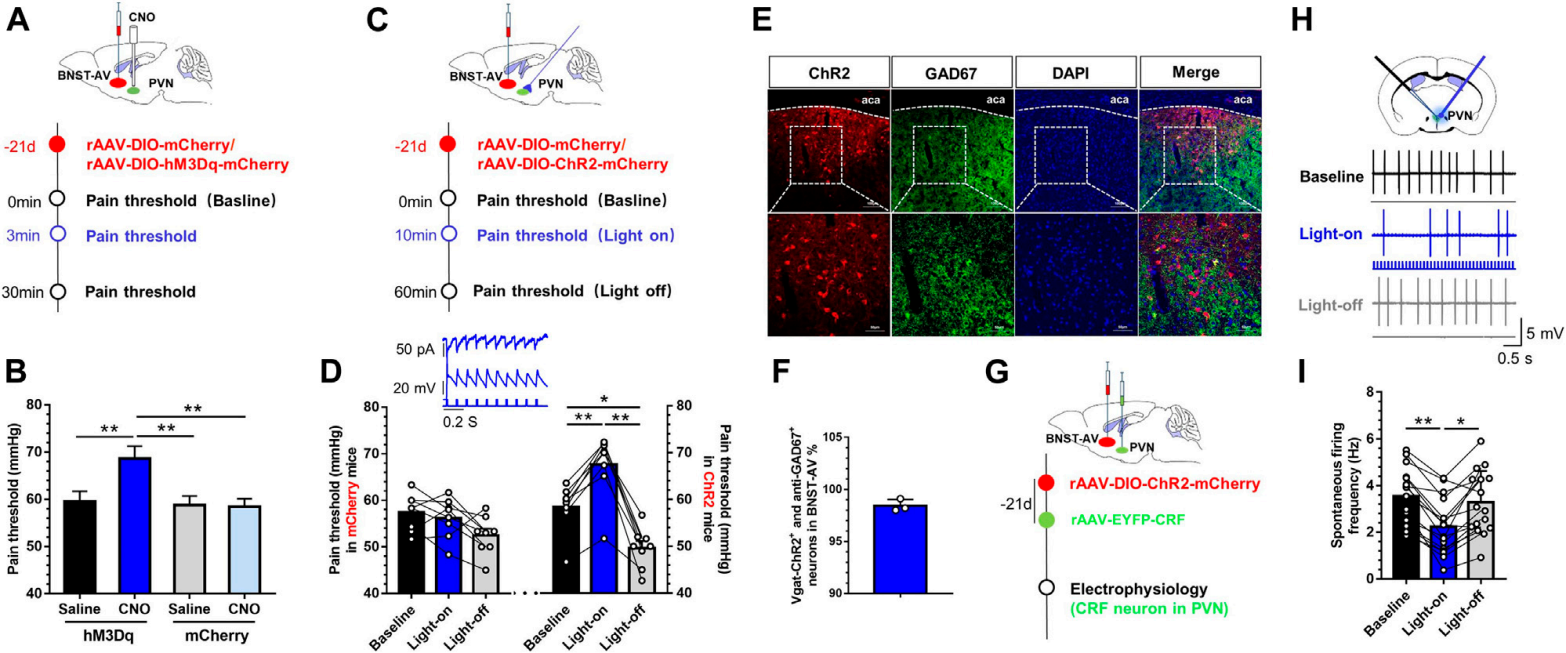
Activation of GABAergic BNST_AV_-PVN neurons alleviated the visceral pain in mice experiencing MS **(A)** Schema for injection of AAV-DIO-hM3Dq-mCherry into the BNST-AV for rescue of inhibitory neurons in Vgat-Cre mice experiencing MS **(B)** The visceral pain threshold significantly increased post-CNO PVN-injection (*n* = 6, **p < 0.01) **(C)** Schematic diagram of AAV-DIO-ChR2-mCherry injection into the BNST-AV and fiber-optic incannulation into the PVN of Vgat-Cre mice **(D)** The visceral pain threshold was significantly increased after photoactivation of the terminal of PVN-projecting ChR2-mCherry-positive neurons from BNST-AV area (*n* = 6, **p < 0.01), and optogenetic stimulation to GABA neurons in BNST-AV region led to laser-dependent hyperpolarization current (473nm laser, wave width: 10ms, frequency: 10Hz) **(E)** The Vgat-ChR2-mCherry neurons were stained with antibody to GAD67 to verify effects of virus-labeled GABAergic neurons in the BNST-AV **(F)** The co-standard rate of labeled GABAergic neurons and anti-GAD67 protein was 98.4% **(G)** *In vitro* electrophysiological recordings were conducted in ChR2-injected mice **(H)** The schema of spontaneous firing frequency in CRF neurons of PVN **(I)** Photoactivation of the terminal of PVN-projecting eNpHR-mCherry-positive neurons from BNST-AV area decreased the firing rate of CRF neurons in PVN from brain slice from MS mice (*n* = 18, six mice per group; **p < 0.01).

Ultimately, we adopted identical chemogenetic and optogenetic approaches to manipulate GABAergic BNST_AV_-PVN neurons in a cohort of Vgat-Cre mice with a history of MS. Mice received AAV-DIO-hM3Dq-mCherry injection as did hM4Di (Figure 6A). MS mice exhibited significantly attenuated visceral pain after PVN-injection of CNO compared with the vehicle group (Figure 6B), suggesting that MS induced long-term GABAergic BNST_AV_-PVN inhibition. One-way ANOVA showed a significant difference [F (3,20) = 31.99, *p* < 0.01; Figure 6B]. Post hoc Bonferroni multiple comparisons showed a significant increase in the visceral pain threshold compared with other three groups (*p* < 0.01). Thereafter, we expressed excitatory Cre-dependent channelrhodopsin-2 (rAAV-Ef1-DIO-hChR2(H134R)-mCherry-WPRE-Pa) in the BNST-AV GABAergic neurons and monitored the visceral pain threshold during photo-stimulation of synaptic terminals. The experimental flow of virus injection was shown in Figure 6C. The visceral pain threshold was increased and pain was relieved under light-on condition in ChR2 group compared with vehicle group [F (2,21) = 20.89, *p* < 0.01; Figure 6D]. As illustrated in Figure 6E, the double immunofluorescence of ChR2 virus and anti-GAD67 protein. 98.4% ChR2-tagged Vgat neurons were GAD67-inmmue-positive (Figure 6F). Figure 6G depicted the combined technique with specific electrophysiological recording of CRF neurons in PVN. The schema of discharge frequency was presented in Figure 6H. The discharge frequency of CRF neurons in PVN was decreased compared with the profiles before and after light switching-on [F (2,45) = 4.94, *p* < 0.01; Figure 6I]. These findings further authenticated the pivotal role of PVN-projecting GABAergic neurons in BNST-AV region in the modulation of visceral hypersensitivity.

## Discussion

In this study, we characterized an MS model generated in Vgat-Cre mice. The results supported the hypothesis that MS can produce striking visceral hypersensitivity with concomitant activation of CRF neurons in PVN. Presynaptic innervation of PVN CRF neurons, as determined by lumafluor retrobeads and retrograde virus, was markedly localized in BNST-AV region. Further, ablation/inhibition of GABAergic BNST_AV_-PVN neurons via AAV-Casp3/AAV-hM4Di/AAV-eNpHR precipitated the activation of PVN CRF neurons and visceral hypersensitivity. Moreover, stimulation of GABAergic BNST_AV_-PVN neurons by means of AAV-hM3Dq/AAV-ChR2 decreased the activities of PVN CRF neurons and alleviated the visceral hypersensitivity. These results implied that inhibition of GABA neurons in the BNST-AV region projecting to CRF neurons in PVN facilitated the development of visceral hypersensitivity. This study may yield a novel insight into the understanding of the circuit mechanism of visceral hypersensitivity as manifested in patients with IBS and provide a potential therapeutic target for IBS treatment.

In the present study, animal model of visceral hypersensitivity was established via neonatal MS in Vgat-Cre mice. Stress or an adverse event in the early life can potentially precipitate the visceral hypersensitivity and pain. Functional magnetic resonance imaging also demonstrates that perception of increased visceral hypersensitivity in IBS patients may benefit central management of pain (Flak et al., 2009). Consequently, visceral hypersensitivity is a key contributor of both brain and gut psychopathology as well as their underlying reciprocity (Senba and Ueyama, 1997). Further, ELS experience may contribute to persistent modifications of neurocircuitry, neuronal plasticity, functionality, and increase susceptibility to the development of both pain-relevant behavior profiles and psychiatric morbidities in patients with IBS (O'Mahony et al., 2017; Rincel et al., 2019; Zhu et al., 2017). Thus, MS in rodents is a well-validated model of ELS in mimicking IBS in humans (Gunn et al., 2013). A large body of literature has established mechanisms by which MS can impact the development of brain and the stress systems of the body, including visceral hypersensitivity, hyper-responsiveness of the HPA axis, peripheral motility abnormalities, altered intestinal permeability, etc. (Oines et al., 2012; Rincel and Darnaudery, 2020).

In line with previous reports (Fuentes and Christianson, 2018; Tang et al., 2017), we confirmed that MS Vgat-Cre mice presented pronounced visceral hypersensitivity and anxiety- and depression-like behaviors in adulthood, coupled with upregulation of CRF protein expression and activation of CRF neurons in PVN (Figures 1, 2). The PVN has the densest distribution of CRF expression, and the CRF cell density in PVN was larger than 20000 cells/mm^3^ (Peng et al., 2017). CRF + neurons account for 33.12% of PVN neurosecretory neurons (Simmons and Swanson 2009; Russell 2018). These CRF neurons are thought to be primarily glutamatergic but also partially GABAergic (Dabrowska et al., 2013). They receive inputs from various brain regions and send projections to the median eminence. The presence of GABA with certain CRF-containing neurons of the PVN projecting to the median eminence may terminate the CRF action (Meister et al., 1988). Moreover, GABA is reported to be a dominant inhibitory neurotransmitter in the PVN. PVN administration of exogenous GABA (0.3mM, 0.2µl) alleviated the visceral hypersensitivity in mice. As per Schmidt M’s report (Schmidt et al., 2001), we designated 3µM as the standard GABA concentration in the electrophysiology. Exogenous GABA inhibited the discharge frequency of CRF neurons in PVN in mice with neonatal MS. CRF neurons integrate both external and visceral stress-responsive information, hence regulating neuroendocrine, autonomic and cognitive and emotional outcomes (Jiang et al., 2019), etc. Besides the local influences on the stress responses, GABA in the PVN decreases sympathetic outflow and blood pressure, and inhibits the adipose afferent reflex that promotes lipolysis and energy expenditure (Ding et al., 2015). These GABAergic mechanisms in the PVN are important for the physiological integration. The activation of PVN CRF neurons might have been mediated by a variety of mechanisms, including the reduction of DNA methylation in epigenetics, early exposure to high concentrations of glucocorticoid and glucocorticoid receptor in the brain, multiple inflammatory factors, the modifications of the nervous system and neuroendocrine variations in the CNS (Heim et al., 1997), etc. Therein, BNST is known to receive direct limbic or cortical input and to intimately innervate the PVN. BNST exhibits sexual dimorphism due to its neuroanatomical connectivity and neurochemical property, the consensus regarding the characteristics of IBS. These findings premise our interest with respect to the neurocircuitry between the BNST and PVN neurons.

Here, we designated the Vgat-Cre mice on the grounds that the Cre-Lox system has provided the optimal target of genetically defined neurons via virus combination. Tracing of Lumafluor retrobeads showed that PVN received projection from BNST-AV, BNST-AL, BNST-AM, mPFC, DR, etc. Moreover, the red fluorescence was detected mainly in BNST-AV region 21 days after PVN microinjection of retrograde DIO-mCherry, which might be attributed to the optimal anatomical proximity and tight functional connections. Furthermore, MS mice presented downregulated expression of GAD65 and GAD67 proteins in BNST-AV region and decreased discharge frequency of GABA neurons (Figure 3). As is well acknowledged, BNST-AV is composed of over 90% GABAergic neurons, and reduced GABAergic inhibition would result in the activation of PVN-projecting neurons. Furthermore, the subnucleus inter-connections of BNST are complicated by their asymmetry or reciprocity (Gungor et al., 2018). However, BNST-AL and BNST-AM gradually become smaller and fused to the ventral region at the anterior commissure. Accordingly, the anterior ventral region of BNST has been postulated to integrate the internal information and post-output to PVN (Walker et al., 2003).

Subsequently, in order to elucidate whether GABAergic BNST_AV_-PVN neurons mediate visceral hypersensitivity in Vgat-Cre mice, we manipulated these neurons by means of apoptotic virus, chemogenetic and optogenetic approaches. Ablation of BNST-AV GABAergic neurons by means of Casp3-apoptotic virus induced the activation of CRF neurons in PVN and exacerbated the visceral pain in normal mice (Figure 4). Likewise, Cre-dependent viral infection of hM4Di-mCherry or eNpHR-mCherry in BNST-AV (PVN CNO-administration or light-stimulation) induced the decreased visceral pain threshold and visceral hypersensitivity precipitated by inhibition of GABAergic BNST_AV_-PVN circuit in normal mice (Figure 5). By consensus, selective ablation of the posterior BNST or GABAergic anterior BNST neurons leads to stress-induced HPA axis activation and increased expression of c-Fos in PVN, suggesting that GABAergic neurons in BNST plays a vital role in suppressing the HPA axis in stress (Choi et al., 2008; Radley et al., 2009). However, Choi et al. reported that the anteroventral BNST lesions are involved in the inhibition of the HPA axis (Choi et al., 2007). Albeit the majority of neurons projecting to PVN are GABAergic, sporadic glutamatergic neurons of approximately 1–3% in BNST-AV region might contribute to the inhibitory effect (Csáki et al., 2000). Paradoxically, photoexcitation of PVN-innervating, ChR2-expressing GABAergic terminals from BNST-AV did enhance the visceral pain threshold and decrease the spontaneous discharge frequency of CRF neurons in PVN in MS mice, as well as the hM3Dq-expressing GABA terminals by chemogenetics (Figure 6). These findings are compatible with the hypothesis that dysfunction of GABAergic BNST_AV_-PVN circuit might participate in the visceral hypersensitivity induced by neonatal colorectal distension in SD rats (Song et al., 2020).

In summary, our study demonstrated that Vgat-Cre mice subjected to MS may render the persistent visceral hypersensitivity-related dysfunction of GABAergic BNST_AV_-PVN circuit, ultimately resulting in the activation of CRF neurons in PVN. Our findings provide circuit-based approaches to higher precision in defining the subregions and cell typology in visceral pain, highlighting the therapeutic orientation towards which manipulation of the GABAergic BNST_AV_-PVN pathway could be a perspective for visceral pain.

## Conclusions

In conclusion, we provided evidence that excitation of BNST-AV neurons could modulate the activity of CRF neurons in PVN in mice susceptible to visceral hypersensitivity. Dysfunction of GABAergic BNST_AV_-PVN circuit predisposed the mice with neonatal MS to the disinhibition of PVN CRF neurons and the development of visceral hypersensitivity, thus validating the involvement of BNST_AV_^GABA^-PVN^CRF^ circuit in the regulation of visceral hypersensitivity. A priori, our present findings provide a potential neurocircuitry basis for therapeutic interventions in chronic visceral pain.

## Data Availability

The original contributions presented in the study are included in the article/Supplementary Material, further inquiries can be directed to the corresponding author.
